# Case Report: Para-infectious cranial nerve palsy after bacterial meningitis

**DOI:** 10.3389/fimmu.2022.1000912

**Published:** 2022-10-06

**Authors:** Giovanni Zanotelli, Lorenzo Bresciani, Mariagiulia Anglani, Alessandro Miscioscia, Francesca Rinaldi, Marco Puthenparampil

**Affiliations:** ^1^ Neurology Unit, Azienda Ospedaliera di Padova, Padova, Italy; ^2^ Department of Neuroscience, Università degli Studi di Padova, Padova, Italy; ^3^ Neuroradiology Unit, Azienda Ospedaliera di Padova, Padova, Italy

**Keywords:** facial palsy, parainfectious disease, bacterial meningitis, parainfectious cranial nerve palsy, *Neisseria meningitidis*

## Abstract

A 27-year-old woman was admitted to our hospital for fever, associated with headache, nausea, and vomiting, and she rapidly developed mild left facial nerve palsy and diplopia. Neurological examination revealed mild meningitis associated with bilateral VI cranial nerve palsy and mild left facial palsy. As central nervous system (CNS) infection was suspected, a diagnostic lumbar puncture was performed, which revealed 1,677 cells/μl, 70% of which were *polymorphonuclear* leukocytes. Moreover, multiplex PCR immunoassay was positive for *Neisseria meningitidis*, supporting the diagnosis of bacterial meningitis. Finally, IgG oligoclonal bands (IgGOB) were absent in serum and cerebrospinal fluid (CSF). Therefore, ceftriaxone antibiotic therapy was started, and in the following days, the patient’s signs and symptoms improved, with complete remission of diplopia and meningeal signs within a week. On the contrary, left facial nerve palsy progressively worsened into a severe bilateral deficit. A second lumbar puncture was therefore performed: the CSF analysis revealed a remarkable decrease of pleocytosis with a qualitative modification (only lymphocytes), and oligoclonal IgG bands were present. A new brain MRI was performed, showing a bilateral gadolinium enhancement of the intrameatal VII and VIII cranial nerves bilaterally. Due to suspicion of para-infectious etiology, the patient was treated with oral steroid (prednisolone 1 mg/kg/day), with a progressive and complete regression of the symptoms. We suggest that in this case, after a pathogen-driven immunological response (characterized by relevant CSF mixed pleocytosis and no evidence of IgGOB), a para-infectious adaptive immunity-driven reaction (with mild lymphocyte pleocytosis and pattern III IgGOB) against VII and VIII cranial nerves started. Indeed, steroid administration caused a rapid and complete restoration of cranial nerve function.

## Introduction

*Neisseria meningitidis* is a Gram-negative diplococcus that can cause invasive meningococcal disease, such as bacterial meningitis. It is a member of the normal nasopharyngeal microbiome, and it can spread *via* aerosol or through oral and nasal secretions (1). Surveillance data from 25 European countries during 2004–2014 showed an annual incidence of invasive meningococcal disease ranging from 0.3 and 2.9 cases per 100,000, with a significantly decreasing annual trend in most countries (2). Nevertheless, *N. meningitidis* is associated with substantial mortality (5%–10%) and severe permanent disabilities, such as cognitive defects, hearing loss, vision deficits, and epilepsy (3–5).

## Case description

**Figure 1:**
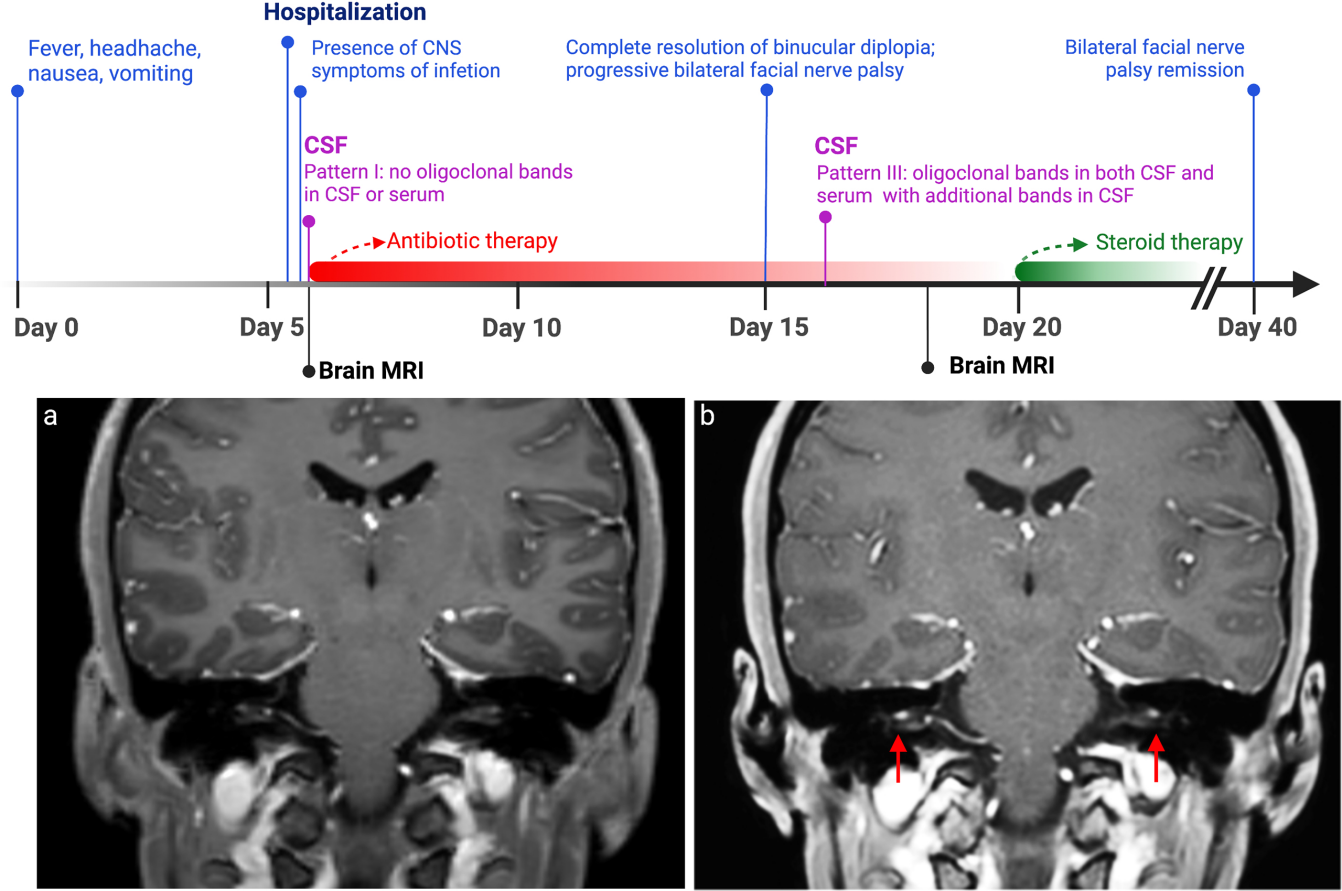
Disease timeline. In this timeline are displayed clinical (shown in blue) and CSF (in purple) changes in relation to antibiotic course (in red) and steroid therapy (in green). On the bottom, MRI T1-weighted imaging with gadolinium contrast performed after 18 days from clinical onset **(A)** demonstrated bilateral pathologically relevant gadolinium enhancement in VII and VIII cranial nerves’ intrameatal tract bilaterally (red arrows), which was not present in the first MRI **(B)**. CSF, cerebrospinal fluid.

A 27-year-old Italian woman living in London who returned to Italy recently suddenly presented fever, sore throat, headache, nausea, vomiting, and transient binocular diplopia (for the disease timeline see **Figure 1**). After 6 days, she went to the emergency department. At the first evaluation, she had no alteration of consciousness, and her vital signs were normal. Her previous medical history was unremarkable except for increased activity levels of coagulation factors (factor II, IX, and X) and vaccination for meningococcus C in 2009 and SARS-CoV-2 in August 2021 (two doses of RNA-based vaccine). Laboratory findings included increased leukocytes count (29,010 cells/μl), neutrophils especially (25,670 cells/μl), and a remarkable C-reactive protein (CRP) (390 mg/dl) and procalcitonin (2.70 ng/ml) increase. Neurological examination was normal, with no evidence of symptoms reflecting meningeal irritation. The head CT scan was normal. Therefore, the patient was admitted to an internal medicine division. However, the following day (7 days after the first symptom suggestive of infection), the patient rapidly developed progressive diplopia with evidence of bilateral abducens nerve palsy, mild left facial nerve palsy with involvement of both superior and inferior branches, nuchal rigidity, and *Lasègue’s sign.* As meningitis was suspected, a diagnostic lumbar puncture was performed. At visual inspection, the collected cerebrospinal fluid (CSF) wasturbid and *cloudy*. Standard analysis showed the presence of pleocytosis (1,677 cells/μl, 70% of which were *polymorphonuclear* leukocytes), increased protein (0.90 g/L) and lactate concentrations (6.0 mmol/L), and decreased CSF glucose concentration (1.2 mmol/L). The diagnosis was bacterial meningitis, and antibiotic therapy based on ceftriaxone 4 g/day was started after the admission to our neurology unit. Based on the multiplex *PCR* immunoassay for the rapid diagnosis of infectious meningitis, which provided results in a few hours and showed the presence of *N. meningitidis* W135 serogroup, antibiotic therapy was confirmed. Isoelectric focusing did not reveal any IgG oligoclonal band (Pattern I). In the next few days, the patient’s signs and symptoms started to rapidly improve, with complete remission of diplopia and meningeal signs within a week. Also, blood cultures resulted positive for *N. meningitidis*, further confirming the infection.

Brain magnetic resonance imaging (brain MRI) with gadolinium contrast was performed, with evidence of leptomeningeal T2/fluid-attenuated inversion recovery (FLAIR) signal hyperintensities at the level of transverse sinuses, with concurrent gadolinium-based enhancement at diffusion-weighted imaging (DWI) sequences; these findings were compatible with the presence of leptomeningeal purulent material. No cranial nerve enhancement was observed.

**Figure 2:**
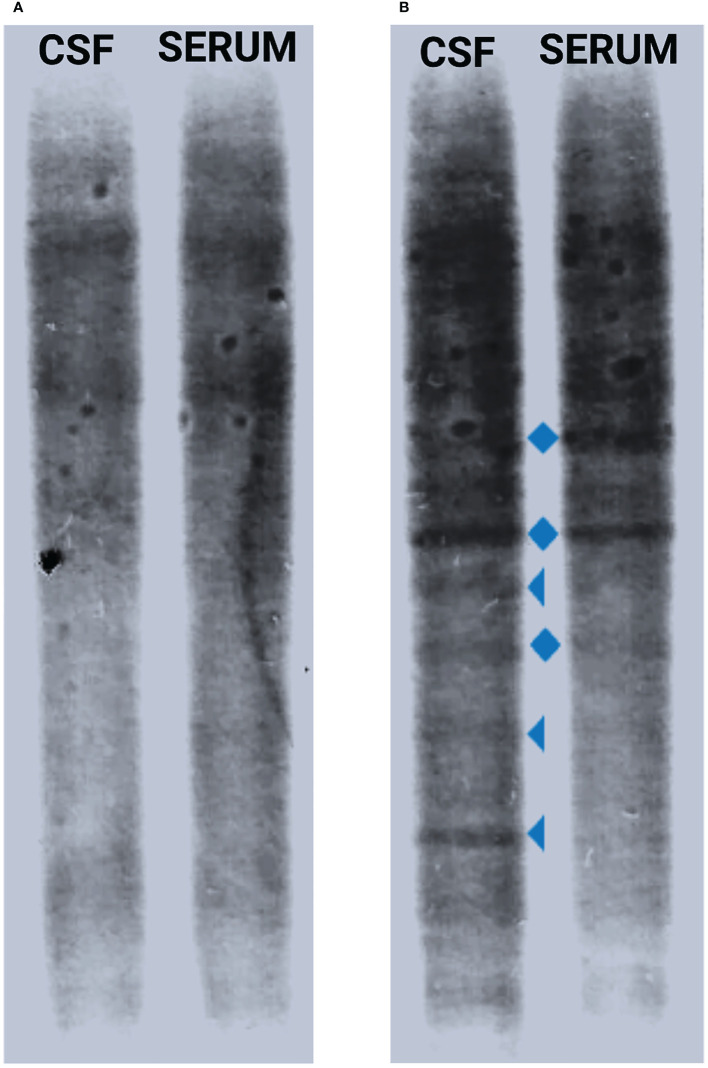
CSF IgGOB pattern modification. When the first lumbar puncture was performed at the beginning of the infection, no oligoclonal bands in CSF or serum were found (pattern I) **(A)**, while after 11 days of antibiotic therapy, there was presence of a few CSF-restricted oligoclonal bands (triangle) together with mirror serum–CSF bands (diamond) (pattern III) **(B)**. CSF, cerebrospinal fluid; IgGOB, IgG oligoclonal bands.

However, after the complete remission, left facial nerve palsy progressively worsened into a severe bilateral deficit. No serum antibodies against GM1, GM2, GD1a, GD1b, or GQ1b were detected. A second lumbar puncture was therefore performed on day 10 of hospitalization: the CSF was clear, and its analysis revealed a remarkable decrease of pleocytosis (103 cells/μl) with a qualitative modification (only lymphocytes) and reduced amount of protein (0.75 mg/dl). Interestingly, oligoclonal IgG bands (pattern III: oligoclonal IgG in CSF with additional identical bands in CSF and serum) were present (**Figure 2**). A follow-up brain MRI with gadolinium contrast was performed 11 days after the first MRI scan: a reduction of the hyperintensity of the leptomeninges at the level of the transverse sinuses and bilateral pathologically relevant gadolinium enhancement in VII and VIII cranial nerves’ intrameatal tract bilaterally were observed. As a para-infectious process was suspected, oral steroid therapy was started based on prednisolone 1 mg/kg/day (weight 50 kg) for 5 days, followed by a subsequent decalage for the next 2 weeks, with complete and persistent regression of the symptomatology. Follow-up brain MRI with gadolinium contrast after 4 months showed no facial nerve abnormalities, and the patient did not develop any additional symptoms.

## Discussion

The clinical onset of the patient’s disease was quite slow and benign for *N. meningitidis*, which typically develops very rapidly. However, the initial presentation of meningococcal disease with a flu-like illness and prodromal symptoms (such as fever, headache, and respiratory and gastrointestinal symptoms) is often reported (6). Cranial nerve palsies during acute bacterial meningitis are not uncommon, as they can be present at the onset or during the infection in 9%–12% of adult patients (7–9). Particularly, oculomotor, abducens, facial, and glossopharyngeal cranial nerve disorders are often observed (7). Nevertheless, cranial nerve palsy in *N. meningitidis* infections is rarely described, and, to our knowledge, only a few cases in adult patients and one pediatric case have been reported in recent times (10–13).

The pathological basis for cranial nerve palsies is not always clear, but it is believed that it might be determined by either the increased intracranial pressure or the meningeal inflammatory reaction adjacent to the cranial nerves (5). In our case herein reported, we first observed a bilateral abducens nerve palsy and associated binocular diplopia, present from the beginning of the infection, and improved when antibiotic therapy was initiated, suggesting a cause directly related to *Neisseria* infection.

The absence of any pathological findings along the VI cranial nerve observed by the MRI might suggest that its deficit might be determined by intracranial hypertension. On the contrary, bilateral facial nerve palsy developed with different characteristics. First, from a radiological point of view, the first MRI with gadolinium contrast performed at the beginning of the infection did not show T2/FLAIR signal intensities or contrast enhancement of the seventh cranial nerves, while pathological gadolinium enhancement was observed only after about 2 weeks from the clinical onset when the infectious process was almost remitted. The normal facial nerve can show various enhancement patterns in MRI sequences; however, intense enhancement of the intrameatal segment, as we observed in our case, can be considered pathological and is often described in Bell’s palsy, owing to the breakdown of blood–peripheral nerve barrier and the subsequent diffusion of contrast into the endoneurial space (14).

Moreover, from an immunological point of view, oligoclonal bands were observed in the second analysis of the cerebrospinal fluid, while they were completely absent in the analysis performed in the diagnostic phase 9 days earlier. The presence of identical oligoclonal IgG bands in CSF and serum may be a common finding in acute bacterial meningitis, as a consequence of the systemic immune response; however, oligoclonal IgG band patterns suggestive of intrathecal immunoglobulin production are less common (15), although they are well characterized in chronic bacterial meningitis, such as lymphocytic meningoradiculitis due to *Borrelia burgdorferi* (16) and tuberculosis meningitis (17).

Finally, from a therapeutic point of view, although a mild left facial nerve deficit was already present during admission to our unit, it gradually worsened after ceftriaxone administration, with progression in severe left palsy and development of contralateral facial nerve deficit. Eventually, the bilateral deficit rapidly improved with oral steroid therapy, with complete remission of the symptomatology.

The Infectious Diseases Society of America (IDSA) guidelines recommend the use of dexamethasone (0.15 mg/kg) for 2–4 days with the first dose administered 10–20 min before, or at least concomitant with, the first dose of antimicrobial therapy in adults with suspected acute bacterial meningitis. The use of corticosteroid therapy administered in the initial phase of infection showed a lower rate of neurological sequelae in adult patients (i.e., seizures and focal neurological deficits). Also, it is associated with reduced mortality in *Streptococcus pneumoniae* meningitis, but not in *N. meningitidis* or *Haemophilus influenzae* meningitis (18). In our case, the patient did not receive any steroids before starting antibiotic therapy. Although the use of corticosteroids in the early phase of the disease, through the downregulation of pro-inflammatory cytokine production, may be effective in decreasing pathophysiologic consequences of the inflammation induced by bacterial meningitis (blood–brain barrier (BBB) permeability increase, which determines cerebral edema, intracranial hypertension, and neuronal injury) (19), the effect on BBB might limit or slow immunity recruitment within the CNS. In our case, since neurological symptoms rapidly improved, we decided to delay steroid administration. Despite that there was no evidence that the para-infectious mechanism would be prevented by steroid administration, we cannot exclude that in our case an early administration of dexamethasone would have prevented secondary transient autoimmune reactivity.

Taking into account all those findings, we believe that the bilateral involvement of the facial cranial nerve and the late clinical progression of its deficit may have been not directly related to meningeal inflammation or other acute infectious causes but to some indirect para-infectious inflammatory process.

Cranial nerve neuritis has been described as a rare para-infectious manifestation of viral diseases (20–22). To our knowledge, our case could be the first report of para-infectious cranial nerve palsy associated with acute bacterial meningitis. Interestingly, a full recovery from bilateral facial nerve palsy was achieved with oral steroid therapy, which exerts its effects through immunosuppressive action, suggesting a possible role of the immune response in the development of the nerve deficit, as the oligoclonal IgG bands’ finding may also indicate. Since steroid administration in bacterial meningitis might prevent also para-infectious central and peripheral nervous system involvement, our case report suggests that steroid administration also rapidly improves meningitis.

## Data availability statement

The original contributions presented in the study are included in the article/supplementary material. Further inquiries can be directed to the corresponding author.

## Ethics statement

Written informed consent was obtained from the individual for the publication of this case report. All potentially identifiable images or data included in this article have been anonymized.

## Author contributions

ZG and BL acquired clinical and radiological data and wrote the first draft of the manuscript. AM revised all the MRI sequences, radiologically followed up the patient, and wrote the manuscript. MA, RF, and PM supervised the clinical and therapeutic approach, conceptualized the manuscript, and wrote the final draft. All authors contributed to the article and approved the submitted version.

## Conflict of interest

The authors declare that the research was conducted in the absence of any commercial or financial relationships that could be construed as a potential conflict of interest.

## Publisher’s note

All claims expressed in this article are solely those of the authors and do not necessarily represent those of their affiliated organizations, or those of the publisher, the editors and the reviewers. Any product that may be evaluated in this article, or claim that may be made by its manufacturer, is not guaranteed or endorsed by the publisher.

## References

[1] Hollingshead S Tang CM . An overview of neisseria meningitidis. Methods Mol Biol (2019) 1969:1–16. doi: 10.1007/978-1-4939-9202-7_1 30877666

[2] Whittaker R Dias JG Ramliden M Ködmön C Economopoulou A Beer N . ECDC network members for invasive meningococcal disease. The epidemiology of invasive meningococcal disease in EU/EEA countries, 2004-2014. Vaccine (2017) 35(16):2034–41. doi: 10.1016/j.vaccine.2017.03.007 28314560

[3] Young N Thomas M . Meningitis in adults: diagnosis and management. Intern Med J (2018) 48(11):1294–307. doi: 10.1111/imj.14102 30387309

[4] Heckenberg SGB de Gans J Brouwer MC Weisfelt M Piet JR Spanjaard L . Clinical features, outcome, and meningococcal genotype in 258 adults with meningococcal meningitis: a prospective cohort study. Med (Baltimore) (2008) 87(4):185–92. doi: 10.1097/MD.0b013e318180a6b4 18626301

[5] Durand ML Calderwood SB Weber DJ Miller SI Southwick FS Caviness VS Jr . Acute bacterial meningitis in adults. A review of 493 episodes. N Engl J Med (1993) 328(1):21–8. doi: 10.1056/NEJM199301073280104 8416268

[6] Johri S Gorthi SP Anand AC . Meningococcal meningitis. Med J Armed Forces India (2005) 61(4):369–74. doi: 10.1016/S0377-1237(05)80071-1 PMC4922928 27407812

[7] Fuentes-Antrás J Ramírez-Torres M Osorio-Martínez E Lorente M Lorenzo-Almorós A Lorenzo O . Acute community-acquired bacterial meningitis: Update on clinical presentation and prognostic factors. New Microbiol (2019) 41(4):81–7. 30994177

[8] Bijlsma MW Brouwer MC Kasanmoentalib ES Kloek AT Lucas MJ Tanck MW . Community-acquired bacterial meningitis in adults in the Netherlands, 2006-14: A prospective cohort study. Lancet Infect Dis (2016) 16(3):339–47. doi: 10.1016/S1473-3099(15)00430-2 26652862

[9] de Gans J van de Beek D Investigators EDiABMS . Dexamethasone in adults with bacterial meningitis. N Engl J Med (2002) 347(20):1549–56. doi: 10.1056/NEJMoa021334 12432041

[10] Senda J Adachi T Tago M Mori M Imai H Ogawa Y . Acute bilateral oculomotor nerve palsy in an adult patient with neisseria meningitidis. Intern Med (2019) 58(11):1639–42. doi: 10.2169/internalmedicine.2098-18 PMC6599925 30713317

[11] Rockholt M Cervera C . Images in clinical medicine. Hypoglossal nerve palsy during meningococcal meningitis. N Engl J Med (2014) 371(15):e22. doi: 10.1056/NEJMicm1315227 25295510

[12] Camacho Salas A Rojo Conejo P Núñez Enamorado N Simón de Las Heras R . Bilateral abducens nerve palsy as the initial clinical manifestation of meningococcal meningitis. Enferm Infecc Microbiol Clin (2017) 35(6):388–9. 10.1016/j.eimc.2016.10.002 27876190

[13] Chiu CH Lin TY Huang YC . Cranial nerve palsies and cerebral infarction in a young infant with meningococcal meningitis. Scand J Infect Dis (1995) 27(1):75–6. doi: 10.3109/00365549509018977 7784819

[14] Forsberg P Frydén A Link H . Immunoglobulin abnormalities in the cerebrospinal fluid during bacterial meningitis. J Neuroimmunol (1986) 12(4):299–310. doi: 10.1016/0165-5728(86)90036-6 3760156

[15] Martin R Martens U Sticht-Groh V Dörries R Krüger H . Persistent intrathecal secretion of oligoclonal, borrelia burgdorferi-specific IgG in chronic meningoradiculomyelitis. J Neurol (1988) 235(4):229–33. doi: 10.1007/BF00314352 PMC7088014 3373242

[16] Sindic CJ Boucquey D Van Antwerpen MP Baelden MC Laterre C Cocito C . Intrathecal synthesis of anti-mycobacterial antibodies in patients with tuberculous meningitis. an immunoblotting study. J Neurol Neurosurg Psychiatry (1990) 53(8):662–6. doi: 10.1136/jnnp.53.8.662 PMC488167 2120390

[17] Hong HS Yi BH Cha JG Park SJ Kim DH Lee HK . Enhancement pattern of the normal facial nerve at 3.0 T temporal MRI. Br J Radiol (2010) 83(986):118–21. doi: 10.1259/bjr/70067143 PMC3473534 19546177

[18] Brouwer MC McIntyre P Prasad K van de Beek D . Corticosteroids for acute bacterial meningitis. Cochrane Database Syst Rev (2015) 2015(9):CD004405. doi: 10.1002/14651858.CD004405.pub5 PMC6491272 26362566

[19] Tunkel AR Hartman BJ Kaplan SL Kaufman BA Roos KL Scheld WM . Practice guidelines for the management of bacterial meningitis. Clin Infect Dis (2004) 39(9):1267–84. doi: 10.1086/425368 15494903

[20] Erben Y Gonzalez Hofmann C Steinmetz H Ziemann U . Mononeuritis des n.oculomotorius bei infektiöser mononukleose (Isolated neuritis of the oculomotor nerve in infectious mononucleosis). Nervenarzt (2008) 79(4):462–4. doi: 10.1007/s00115-007-2385-y 18058080

[21] Nazir SA Murphy SA Siatkowski RM . Recurrent para-infectious third nerve palsy with cisternal nerve enhancement on MRI. J Neuroophthalmol (2004) 24(1):96–7. doi: 10.1097/00041327-200403000-00033 15206451

[22] Knoflach K Holzapfel E Roser T Rudolph L Paolini M Muenchhoff M . Case report: Unilateral sixth cranial nerve palsy associated with COVID-19 in a 2-year-old child. Front Pediatr (2021) 9:756014. doi: 10.3389/fped.2021.756014 34976891 PMC8718702

